# Norovirus-Mediated Modification of the Translational Landscape via Virus and Host-Induced Cleavage of Translation Initiation Factors*[Fn FN2]

**DOI:** 10.1074/mcp.M116.062448

**Published:** 2017-01-13

**Authors:** Edward Emmott, Frederic Sorgeloos, Sarah L. Caddy, Surender Vashist, Stanislav Sosnovtsev, Richard Lloyd, Kate Heesom, Nicolas Locker, Ian Goodfellow

**Affiliations:** From the ‡Division of Virology, Department of Pathology, University of Cambridge, Addenbrookes Hospital, Hills Road, Cambridge, UK;; §Laboratory of Infectious Diseases, National Institute of Allergy and Infectious Diseases, National Institutes of Health, Bethesda, Maryland;; ¶Department of Molecular Virology and Microbiology, Baylor College of Medicine, One Baylor Plaza, Houston, TX;; ‖Proteomics facility, School of Biochemistry, University of Bristol, Biomedical Sciences Building, University Walk, Bristol, UK;; **Faculty of Health and Medical Sciences, School of Biosciences and Medicine, University of Surrey, Guildford, UK

## Abstract

Noroviruses produce viral RNAs lacking a 5′ cap structure and instead use a virus-encoded viral protein genome-linked (VPg) protein covalently linked to viral RNA to interact with translation initiation factors and drive viral protein synthesis. Norovirus infection results in the induction of the innate response leading to interferon stimulated gene (ISG) transcription. However, the translation of the induced ISG mRNAs is suppressed. A SILAC-based mass spectrometry approach was employed to analyze changes to protein abundance in both whole cell and m7GTP-enriched samples to demonstrate that diminished host mRNA translation correlates with changes to the composition of the eukaryotic initiation factor complex. The suppression of host ISG translation correlates with the activity of the viral protease (NS6) and the activation of cellular caspases leading to the establishment of an apoptotic environment. These results indicate that noroviruses exploit the differences between viral VPg-dependent and cellular cap-dependent translation in order to diminish the host response to infection.

Noroviruses are the causative agent of the majority of human viral gastroenteritis cases in the developed world (1). Globally, they are responsible for an estimated 200,000 deaths in children under the age of five in developing countries, and in developed countries noroviruses are a major burden on national healthcare infrastructure due to closed wards and economic costs (1). Noroviruses are small, single-stranded, positive-sense RNA viruses best known for infecting humans, but several animal-specific noroviruses have also been identified (2, 3).

As members of the *Caliciviridae*, noroviruses use a virus-encoded VPg protein in place of a 5′ cap structure to recruit eukaryotic initiation factors and direct translation of viral RNAs (4–7). The norovirus nonstructural proteins are generated by the cleavage of a large polyprotein by the viral protease NS6 (Fig. 1*A*) (8, 9), whereas the structural proteins VP1 and VP2 are produced from a subgenomic mRNA produced during replication (10). In the case of murine norovirus (MNV), the only norovirus that can undergo efficient replication in cell culture, a single overlapping reading frame encoding a virulence factor is also present within the VP1 coding region (11). Members of the *Norovirus* genus appear distinct from other caliciviruses in that VPg interacts directly with the scaffolding protein eIF4G^1^ (4, 6), with this representing the key interaction for viral translation, rather than the cap-binding protein eIF4E (7, 12, 13), a further departure from the usual cap-dependent mechanism of protein translation. In addition, we have also shown that norovirus infection causes eIF4E phosphorylation, which may lead to the preferential translation of distinct subsets of cellular mRNAs (14). Other viruses utilize discrepancies between cellular and viral translation to either enable more efficient translation of viral mRNA in the presence of vastly more abundant cellular mRNA (15) or to inhibit the translation of cellular mRNA inhibitory to viral infection (16).

Studies using MNV, the most common model for the study of norovirus–host interactions, have also confirmed the essential role that the innate response plays in the regulation of norovirus infection and pathogenesis. MNV was discovered in RAG/STAT1^−/−^ mice (17), and more recent studies have shown that norovirus infection can be controlled and cleared through interferon λ, with interferon α and β protecting the host from systemic infection even in the absence of adaptive immunity (18, 19). Noroviruses are thought to use several mechanisms to combat the immune response to infection (20). These include disrupting protein export by the human norovirus NS1/2 (21, 22) or NS4 proteins (23, 24) as well as diminishing interferon-stimulated gene (ISG) induction through the activity of the MNV virulence factor VF1 protein (11). Despite these mechanisms, the interferon response is activated during both natural infection in humans (25) or in mice infected with MNV (18), leading to the induction of ISG transcription. A recent study suggests that human norovirus, while sensitive to interferon, is a poor inducer of the interferon response *in vitro*, albeit in conditions where viral RNA is transfected into cells leading to a limited number of cells undergoing active viral replication (26). The recent generation of an enteroid system for the growth and culture of human norovirus will enable this to be investigated in more detail (27). Notwithstanding this, we and others have observed that ISG gene induction observed following murine norovirus infection often does not correlate with the resulting levels of the induced protein (11, 28), suggesting a posttranscriptional regulatory mechanism is also involved in the control of the innate response.

Posttranscriptional control of host translation during apoptosis or viral infection has been investigated previously with the primary mechanisms involved utilizing either phosphorylation of eIF2α or the cleavage of translation initiation factors by viral or host enzymes (16). Of note, the related calicivirus, feline calicivirus, reduces cellular translation through the activity of a viral protease in cleaving the cellular protein PABP (29), and the roles of the picornavirus 3C and 2A proteases, or cellular caspases, in cleaving translation initiation factors have been characterized extensively (16, 30). However, the similarities or otherwise underlying this process in MNV had yet to be studied, and no previous attempt at applying quantitative proteomics to the study of viral-mediated modification of host protein synthesis made.

We investigated the posttranscriptional regulation of ISG mRNA translation during norovirus infection. SILAC-based quantitative proteomics was used to identify specific changes to levels or activity of translation initiation factors within norovirus-infected cells. We observed that alterations to the translatome due to norovirus infection were caused by both direct viral and cellular response mechanisms, resulting in the specific reduction in translation of cellular mRNAs. Inhibition of these modifications lead to the restoration of ISG translation and an impact on viral replication, indicating that norovirus infection limits the ability of the innate immune response to combat infection by posttranscriptional regulation of induced mRNAs.

## EXPERIMENTAL PROCEDURES

### 

#### 

##### Cells and Viruses

Murine RAW 264.7 and BV-2 cells were used for infection experiments; human HEK-293T cells were used for the indicated transfection experiments. All infections were performed with murine norovirus strain CW1 (31). All titer calculations were performed by TCID_50_. All infections were performed at high MOI (10 TCID_50_/cell) unless explicitly indicated, in which case a low MOI of 0.01 TCID_50_/cell was used. See Supplemental Experimental Procedures for more details.

##### Cell Lysis and ^35^S-Methionine Labeling

Cells were lysed in radio-immunoprecipitation buffer for analysis of whole-cell extracts. For metabolic labeling experiments utilizing ^35^S-Methionine, medium was replaced with DMEM containing [^35^S]methionine 30 min prior to the indicated time, and the samples harvested 30 min after the indicated time in radio-immunoprecipitation buffer.

##### m7GTP-Sepharose Enrichment and Polysome Profiling

For analysis of the eukaryotic initiation factor (eIF) complex, initiation factors were enriched on m7GTP-Sepharose beads (Jena Biosciences) following lysis in m7GTP lysis buffer as described in Chung *et al.*, 2014. Polysome profiling was accomplished by centrifuging cytoplasmic lysates for 90 min at 200,000 × *g* over a 10–50% sucrose gradient and analyzed using an Isco Fractionator measuring absorbance at 254 nm. For full details, see Supplemental Experimental Procedures.

##### Mass Spectrometry Analysis

Cells were grown in DMEM containing stable-isotope-labeled forms of arginine and lysine for five passages, with labeling confirmed by mass spectrometry. Unlabeled arginine and lysine were used in the “Light” media, R6 (^13^C_6_) and K4 (D_4_) in the “Medium” media, and R10 (^13^C_6_,^15^N_4_) and K8 (^13^C_6_,^15^N_2_), in the “Heavy” media. 10 cm^2^ dishes containing 1 × 10^7^ cells were used as input for both m7GTP experiments and the whole cell lysate experiments, yielding a minimum of 1 mg total protein per dish. Samples were harvested at the indicated time points and combined following either lysis or m7GTP-Sepharose enrichment. These samples were subject to SDS-PAGE electrophoresis and processed by in-gel trypsinization followed by LC-MS/MS analysis on a Orbitrap Velos instrument at the University of Bristol. For the whole cell lysate experiments, the gel lane was cut into 10 slices and each slice subjected to in-gel tryptic digestion using a ProGest automated digestion unit (Digilab UK). For the m7GTP pulldown experiments, the samples were run into a precast gel and extracted as a single band for tryptic digestion. The resulting peptides were fractionated using an Ultimate 3000 nanoHPLC system in line with an LTQ-Orbitrap Velos mass spectrometer (Thermo Scientific). In brief, peptides in 1% (v/v) formic acid were injected onto an Acclaim PepMap C18 nanotrap column (Thermo Scientific). After washing with 0.5% (v/v) acetonitrile 0.1% (v/v) formic acid, peptides were resolved on a 250 mm × 75 μm Acclaim PepMap C18 reverse phase analytical column (Thermo Scientific) over a 150 min organic gradient, using seven gradient segments (1–6% solvent B over 1 min., 6–15% B over 58 min., 15–32% B over 58 min., 32–40%B over 3 min., 40–90% B over 1 min., held at 90% B for 6 min and then reduced to 1% B over 1 min.) with a flow rate of 300 nl min^−1^. Solvent A was 0.1% formic acid, and Solvent B was aqueous 80% acetonitrile in 0.1% formic acid. Peptides were ionized by nanoelectrospray ionization at 2.1 kV using a stainless steel emitter with an internal diameter of 30 μm (Thermo Scientific) and a capillary temperature of 250 °C. Tandem mass spectra were acquired using an LTQ- Orbitrap Velos mass spectrometer controlled by Xcalibur 2.1 software (Thermo Scientific) and operated in data-dependent acquisition mode. The Orbitrap was set to analyze the survey scans at 60,000 resolution (at *m/z* 400) in the mass range *m/z* 300 to 2000 and the top six multipley charged ions in each duty cycle selected for MS/MS in the LTQ linear ion trap. Charge state filtering, where unassigned precursor ions were not selected for fragmentation, and dynamic exclusion (repeat count, 1; repeat duration, 30 s; exclusion list size, 500) were used. Fragmentation conditions in the LTQ were as follows: normalized collision energy, 40%; activation q, 0.25; activation time 10 ms; and minimum ion selection intensity, 500 counts.

The raw data files were processed and quantified using Maxquant v1.5.5.1 and searched against the Uniprot Mouse database (51,418 entries, dated May 14, 2016) plus a custom fasta file generated in-house containing the MNV-1 (accession DQ285629) protein sequences using the built-in Andromeda search engine (32). Peptide precursor mass tolerance was set at 4.5 ppm, and MS/MS tolerance was set at 0.5 Da. Search criteria included carbaminomethylation of cysteine as a fixed modification. Oxidation of methionine and N-terminal acetylation were selected as variable modifications. Quantification was based on Light (Arg 0, Lys 0), Medium (Arg 6, Lys 4), and Heavy (Arg 10, Lys 8) SILAC labels. Searches were performed with full tryptic digestion, a minimum peptide length of seven amino acids, and a maximum of two missed cleavages were allowed. The reverse database search option was enabled, and the maximum false discovery rate for both peptide and protein identifications was set to 0.01. Quantitation was performed using a mass precision of 2 ppm, and the requantify option in Maxquant was enabled. The presented protein ratios represent the median of the raw measured peptide ratios for each protein. Contaminants (as defined in the default Maxquant list), reverse identifications, and proteins only identified by site were excluded from further analysis. The data were imported into the Perseus software (33) for assigning GO annotations, and pathway analysis of m7GTP proteins showing altered abundance in infected cells was performed using STRING v10.0 (34).

##### Experimental Design and Statistical Rationale

For both whole-cell lysate and m7GTP-Sepharose proteomic experiments, triplicate biological samples were used. 4 h (Medium labeled) or 9 h (Heavy labeled) postinfection samples were compared with a 0 h (Light labeled) control lysate. SILAC label switching (4 h and 9 h swapped) was performed in the second of each set of experiments to control for any effects of the SILAC labeling. Protein ratios were log_2_-transformed and outliers removed by Grubbs outlier detection implemented in Graphpad prism (alpha 0.1). One-way ANOVA was performed to calculate whether the abundance of a protein of interest was altered compared with a control protein (eIF4E) with unaltered abundance in the assay.

##### qRT-PCR

RNA samples were extracted using the Genelute mammalian total RNA extraction kit (Promega) and qRT-PCR analysis performed by the SYBR green method on a Viia 7 instrument. Relative quantification was performed by the ΔΔCt method relative to a GAPDH standard, and absolute quantification was performed by comparing RNA copies to a serially diluted DNA standard.

##### Western Blotting Analysis

Cell lysates were subject to SDS-PAGE electrophoresis and transferred to nitrocellulose membranes according to standard protocols. Blocking and antibody incubation steps were performed in 5% BSA or 5% nonfat dried milk as appropriate for the antibody. Detection was performed using either HRP-conjugated antibodies and chemiluminescent detection or infrared-dye-conjugated secondary antibodies and detection on a Li-Cor Odyssey imager. All densitometry was performed on samples analyzed on a Li-Cor Odyssey imager using the ImageStudioLite software (Li-Cor). Full experimental and antibody details are given in Supplemental Experimental Procedures.

##### Immunofluorescence Microscopy and Puromycylation

Puromycylation was performed as described in (35). In brief, cells grown on glass coverslips were incubated for 5 min in cell culture media supplemented with puromycin and emetine at 37 °C. Cells were transferred to and maintained on ice for subsequent extraction steps. Cells were incubated for 2 min with permeabilization buffer. Cells were then washed once with polysome buffer and fixed with PFA for 15 min at room temperature. PFA was aspirated, PBS was added, and cells were maintained at 4 °C. Subsequent antibody incubations and washing steps followed standard protocols and are described in (36). Imaging was performed on a Leica Sp5 confocal microscopy using a 63x oil objective. Image analysis was performed in the Leica Lita software (Leica Microsystems). Full details are given in Supplemental Experimental Procedures.

##### Accession Numbers

The mass spectrometry proteomics data have been deposited to the ProteomeXchange Consortium via the PRIDE (37) partner repository with the dataset identifiers PXD004984 (whole-cell SILAC experiments) and PXD004983 (m7GTP enrichment).

## RESULTS

### 

#### 

##### Induction of the Innate Immune Response Is a Late Event in Norovirus Infection

**Fig. 1.**
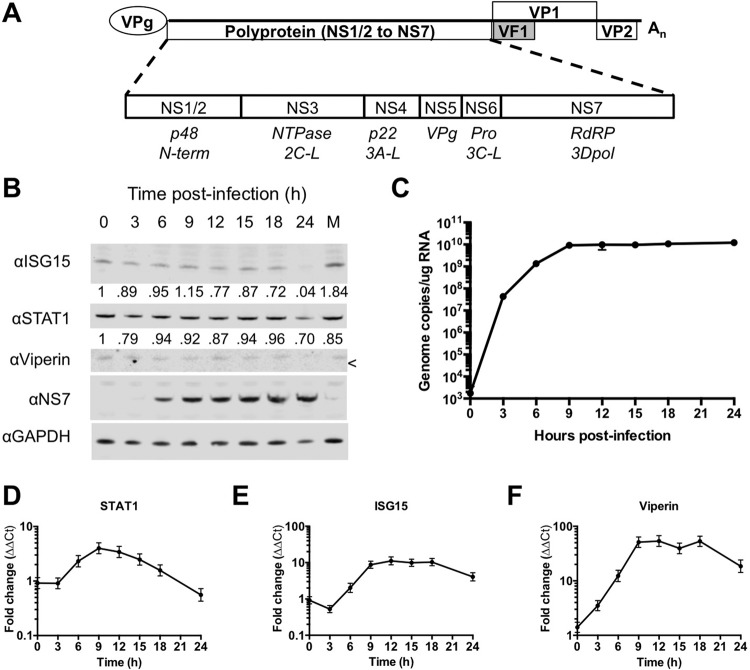
**A defect in ISG protein synthesis, but not mRNA induction is observed during norovirus infection.** (*A*) Genome schematic of the norovirus genome with the four previously described murine norovirus open reading frames are shown (11). The NS1/2–7 nomenclature for the mature peptides generated from ORF1 (described in (8)) is used throughout. (*B*) Western blot for the norovirus NS7 nonstructural protein and the ISGs STAT1, ISG15, and viperin. (*C*) qRT-PCR for norovirus genomic RNA and (*D–F*) for the ISGs STAT1, ISG15, and viperin. The samples for panels (*B–F*) were taken from a high multiplicity of infection (MOI: 10 TCID_50_/cell) timecourse performed in RAW 264.7. The correct position for viperin is indicated with “<” and is immediately beneath the visible nonspecific band. For qRT-PCR *n* ≥ 3, and error bars represent standard deviation.

To examine the kinetics of the induction of the innate response during norovirus infection, we examined the levels of ISG mRNA and proteins produced during highly synchronized infection of immortalized macrophage cells (Figs. 1*B* and 1*C*). A high multiplicity of infection (10 TCID_50_ per cell) was used to ensure synchronous infection and the infection levels were confirmed by immunofluorescence staining (Fig. S1*A*). The levels of representative ISG mRNAs, STAT1, ISG15, and viperin, peaked at around 9 h post infection and with the exception of STAT1 remained at high levels in infected cells (Figs. 1*D*–1*F*). However, levels of the corresponding ISG proteins did not correlate with this increase in RNA levels, with no observable induction of viperin, and no detectable increase in the levels of ISG15 or STAT1 (Fig. 1*B*).

**Fig. 2.**
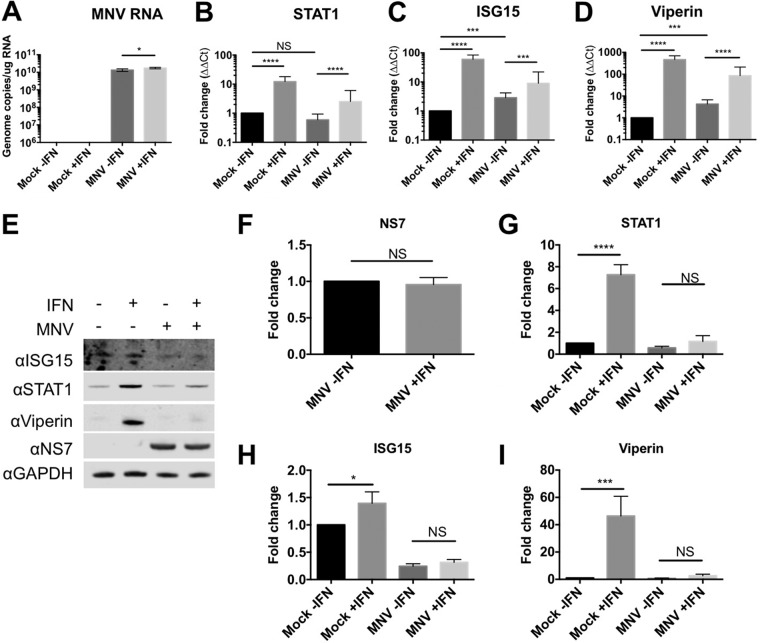
**MNV infected cells respond to interferon at the transcriptional but not posttranscriptional level.** Samples were harvested from RAW 264.7 cells, infected at high MOI (10), 18 h post infection following treatment with interferon or mock culture supernatant. qRT-PCR of norovirus and ISG mRNA levels following interferon treatment (*A–D*) and representative Western blots are shown in (*E*), with densitometry analysis of protein levels in (*F–I*) error bars show standard deviation, *n* = 3. Data are presented relative to a mock, untreated control. Statistical analysis was performed by one-way ANOVA, with the exception of figure (*F*), which was performed by *t* test. For qRT-PCR, statistical analysis was performed on the untransformed ΔΔCt data. (* = <0.05, ** = <0.01, *** = <0.001, **** = <0.0001).

To examine if norovirus replication affected the ability of cells to respond to interferon, we examined the effect of interferon treatment during ongoing norovirus replication (Fig. 2). Treating infected cells with interferon 6 h post infection lead to robust levels of ISG mRNA induction while having a minimal effect on MNV replication (Figs. 2*A*–2*D*). The levels of ISG mRNA produced following IFN treatment of infected cells were slightly, but significantly (*p* ≤ 0.001), decreased when compared with uninfected cells (Figs. 2*A*–2*D*). However, despite robust levels of ISG mRNA transcription, a clear defect in ISG protein production was observed in norovirus infected cells following IFN treatment (Figs. 2*E*–2*I*).

##### Norovirus Infection Leads to a Translational Bias

**Fig. 3.**
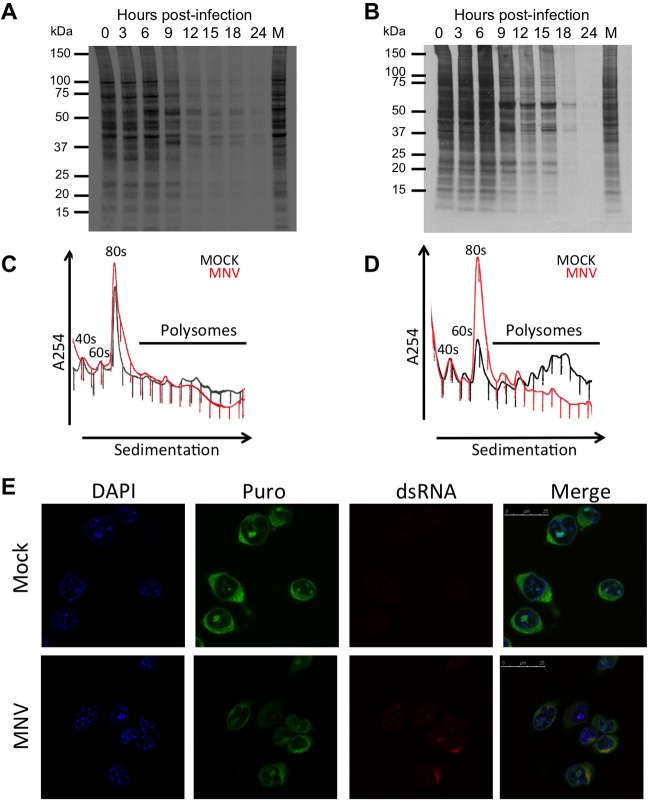
**Norovirus infection alters the translational profile of the host cell.** (*A*) RAW 264.7 or (*B*) BV-2 cells infected at an MOI of 10 TCID_50_/cell with MNV were pulse labeled for 1h at the indicated time points and imaged on a phosphorimager. Polysome profiling of infected (*C*) RAW 264.7 or (*D*) BV-2 cells was performed. (*E*) Puromycylation analysis of infected BV-2 cells shows some minor enrichment of sites of active translation colocalizing with viral replication complexes visualized using anti-dsRNA.

To examine the impact of norovirus infection on global cellular translation, *de novo* protein synthesis was monitored during synchronized infection by ^35^S methionine pulse-labeling. A clear shift in the translation profile was observed in both RAW264.7 (Fig. 3*A*) and BV-2 (Fig. 3*B*) cell lines from 9 h post infection, though this fell short of the host shutoff observed in picornavirus or feline calicivirus infection (29, 38). In line with previous polysome analysis performed on norovirus infected cells (14), a modest loss of polysomes was observed in RAW264.7 cells with a corresponding increase in the 80 s monosome peak (Fig. 3*C*). A much more apparent reduction in polysome formation occurred during norovirus infection of BV-2 cells (Fig. 3*D*). Radio-immunoprecipitation of pulse-labeled proteins from norovirus-infected cells confirmed that viral translation was ongoing while cellular translation is hindered at 12 h post infection (Fig. S1*B*). MNV infection of BV-2 cells follows a similar course to that observed in RAW 264.7 cells (Fig. S1*C*–S1*E*). The loss of polysomes and increase in monosomes is characteristic of a defect in translation initiation (39). Under the conditions used 40 s and 60 s subunits often associate forming RNA-free 80 s monomers, which can be dissociated into free subunits under high salt conditions, while RNA-associated ribosomes remain intact. When polysomes were fractionated under high salt conditions, the 80 s peak dissociated into 40 s and 60 s monomers confirming that the 80 s monomers were not RNA-associated (Figs. S2*A* and S2*B*). A frequently observed mechanism of regulating cellular translation inhibition during viral infection involves the phosphorylation of eIF2α (40). While modest levels of eIF2α phosphorylation were observed during MNV infection (Fig. S2*C*), the kinetics of phosphorylation varied in a cell-type-specific manner and had a poor temporal association with the observed effect on the translation profile, particularly in BV-2 cells. Based on these observations and that eIF2α phosphorylation would also be anticipated to be inhibitory for norovirus translation, which was not in evidence, we concluded that, under the experimental conditions used here, eIF2α phosphorylation did not significantly contribute to the translational bias observed during infection. Studies on pox-virus-infected cells have suggested that the sequestration of sites of active translation to centers of virus replication causes a translational bias (35). This possibility was investigated using puromycylation to visualize active sites of protein synthesis by covalent linkage of puromycin to newly synthesized peptides as described (35). Puromycylation of mock or infected BV-2 cells showed some enrichment of sites of active translation colocalizing with sites of viral RNA replication as determined by staining for dsRNA (Fig. 3*E*). However, this enrichment fell short of the sequestration observed with vaccinia infection (35), with the majority of active translation localizing outside of the viral replication complexes.

##### Quantitative Proteomic Analysis of MNV-Infected Cells and m7GTP-Binding Complexes Reveals Modifications to the eIF Complex

**Fig. 4.**
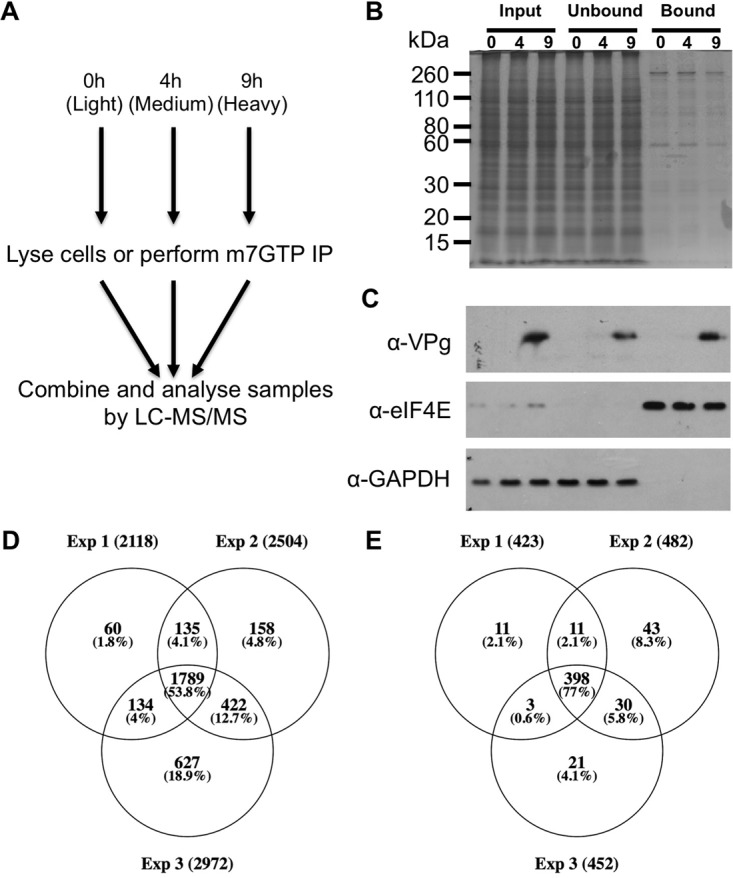
**Quantitative proteomic analysis of translation initiation during MNV infection.** SILAC-based quantitative proteomics was employed to investigate changes to eIF composition during high MOI (10) MNV infection of BV-2 cells. The experimental layout is illustrated in (*A*) with samples taken at mock (0 h) early (4 h) or late (9 h) post infection either lysed or subject to m7GTP-Sepharose purification. A Coomassie gel (*B*) and representative Western blots (*C*) confirm initiation factor enrichment. Venn diagrams illustrating experimental proteome coverage in (*D*) whole-cell lysate, or E) m7GTP-Sepharose pulldowns.

We have previously described the novel mechanism of protein-primed VPg-dependent translation used by caliciviruses (5–7, 12). Given the variation between the initiation factor requirements for host-cell mRNA and viral VPg-dependent RNA translation, a quantitative proteomics approach was used to investigate changes to both the level of relevant translation initiation factors in the host cell, as well as their ability to incorporate into the eIF complex at different times post infection. Using a stable isotope labeling approach (41–43), cells were labeled with light, medium, or heavy stable isotope labelled forms of arginine and lysine. Whole cell lysates were prepared from either mock or infected cells at 4 and 9 h post infection, and these samples used to determine the level of initiation factors within the cell. At the same time, an m7GTP-Sepharose enrichment was performed to determine the effect of viral infection on the composition of eIF4E-containing cap-binding complexes (Fig. 4*A*). Representative Coomassie staining (Fig. 4*B*) and Western blot analysis (Fig. 4*C*) of the purified complex confirmed the enrichment of translation initiation factors, as well as recruitment of the viral VPg protein and loss of a marker for soluble cytoplasmic proteins (GAPDH).

Mass spectrometry analysis of independent biological replicates of the whole cell lysates identified 3,466 proteins with two or more peptides (Fig. 4*D*). As anticipated, the strategy used to enrich the eIF complex resulted in fewer proteins being identified across the three replicates (535); however, a better overlap in the proteins quantified across the replicates (Fig 4*E*). Gene ontology analysis (Table S1) of the m7GTP-Sepharose purified samples performed using STRING (34) revealed that proteins showing an arbitrary ≥2-fold change in their abundance at 9 h post infection were associated with translation (*p* = 4.9E-55), with changes to the ribosomal subunits and the eIF3 complex being particularly significant (*p* = 6.76E-28 to 1.05E-46). GO annotation of both datasets in Perseus revealed that the m7GTP dataset was enriched for proteins with GO terms including the string “translation” (13.6% *versus* 3.8%). The complete proteomics dataset obtained from the analysis of both the whole cell lysate and the m7GTP-enriched complex is provided in Tables S2 and S3. Notably, all viral proteins were identified in the whole-cell dataset and all but NS4 in the m7GTP dataset, including the VF-1 protein/ORF4 alternative reading frame product.

**Fig. 5.**
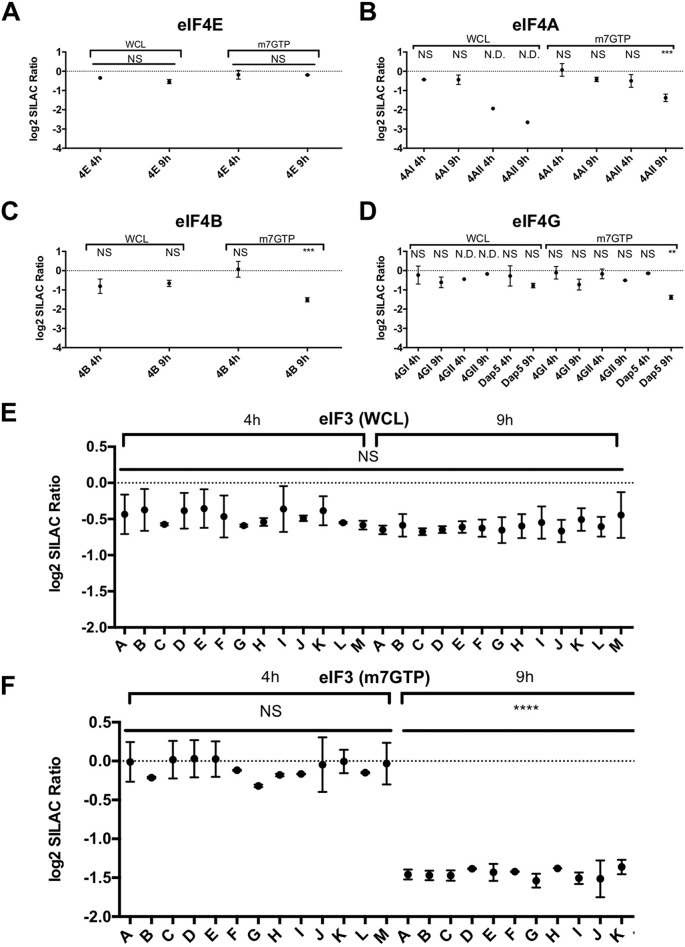
**Norovirus infection alters the abundance and eIF4F association of cellular translation initiation factors.** Mass spectrometry data for individual eIF components identified in the whole cell (WCL) or m7GTP-Sepharose (m7GTP) experiments are shown, including (*A*) eIF4E, (*B*) eIF4A, (*C*) eIF4B, and (*D*) eIF4G. The whole eIF3 complex was successfully identified by mass spectrometry and its relative abundance in (*E*) WCL or (*F*) m7GTP samples is shown. Significance was tested by one-way ANOVA comparing changes to a control protein with unaltered abundance (eIF4E). Changes in eIF4E levels were determined by comparing its 4 h and 9 h levels. Error bars represent standard deviation, * = <0.05, ** = <0.01, *** = <0.001, **** = <0.0001. Where a protein was identified in only a single mass spectrometry replicate, precluding statistical analysis, this is indicated with “N.D.,” otherwise proteins were identified in at least 2/3 replicates.

Alterations to individual eIF components were assessed with no changes in the abundance of eIF4E within infected cells or in its ability to bind to m7GTP-Sepharose being observed (Fig. 5*A*). However, changes to other components of the eIF4F complex were apparent. Reduced levels of the eIF4AII but not eIF4AI isoform of eIF4A were apparent at late time points in cells and in m7GTP-purified complexes (Fig. 5*B*). Another helicase, eIF4B, showed similar levels within whole cell lysates at late times post infection but was reduced within m7GTP-purified complexes (Fig. 5*C*). Isoform-specific variation in the impact of infection on eIF4G levels was also observed with several isoforms showing slight but not significant reductions in cellular abundance and all isoforms showing reduced m7GTP-Sepharose binding, though only the loss of Dap5 was significant (Fig. 5*D*). Please note that due to inconsistency with eIF4GII and Dap5 nomenclature, in this manuscript, eIF4GII (eIF4G3) is used to refer to the ∼170 kDa long form, and Dap5 (eIF4G2) is used to refer to the ∼100 kDa short form, also known as NAT1. As a component of the core of the eIF4F complex, eIF4G interacts with the small ribosomal subunit via the eIF3 complex, which was detected in its entirety in both the whole-cell lysate and in the m7GTP-associated complex (Fig. 5*E* and 5*F*). The abundance of eIF3 components remained largely unaffected during infection (Fig. 5*E*); however, at 9 h post infection, the ability of eIF4F to recruit the eIF3 complex was greatly diminished, consistent with the gene ontology analysis (Fig. 5*F*). Other proteins recruited to the eIF4F complex by eIF3 also showed a similarly reduced ability to bind m7GTP-Sepharose and are detailed in Fig. S3*A*–S3*D*. Western blot analysis of eIF4E-containing complexes by m7GTP-Sepharose enrichment from infected cells confirmed the loss of eIF3D and eIF4GII, as well as the impact of infection on eIF4AII expression (Fig. S3*E*). While large number of proteins showed either unaltered or decreased binding to m7GTP at late times post infection, few showed increased binding, with viral proteins representing the primary exception to this pattern. All viral proteins except NS4 showed enrichment to some extent, including VPg and the protease NS6. Given the previous associations of 3C-like proteases in mediating virus-induced shutoff of host translation via cleavage of initiation factors, the presence of the viral protease NS6 in samples enriched for initiation factors suggested a similar mechanism could be responsible for the alterations to initiation factor complexes observed here.

##### The Norovirus Protease (NS6) Contributes to Translational Inhibition via PABP Cleavage

**Fig. 6.**
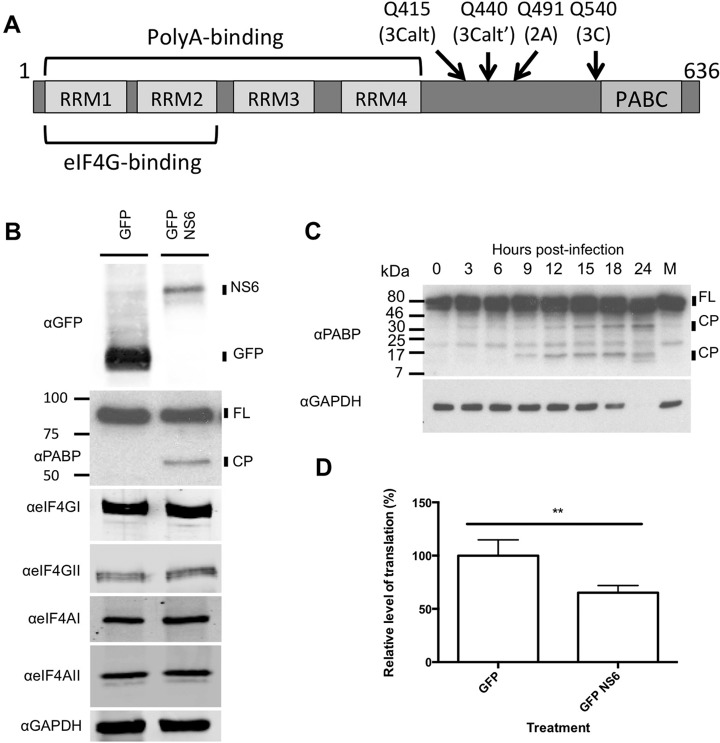
**Cleavage of PABP by the norovirus protease NS6 contributes to reduced cellular translation.** (*A*) Illustration of the domain structure of PABP. (*B*) Western blot analysis of selected eIF proteins in 293T cells transfected with the MNV protease NS6. (*C*) Western blot analysis of PABP cleavage over a MOI 10 infection timecourse in BV-2 cells. (*D*) Analysis of global translation in 293T cells transfected with NS6 assessed by ^35^S-methionine pulse-labeling and quantification on a phosphorimager. Error bars represent standard deviation from three biological replicates. Statistical analysis was performed by one-way ANOVA. (* = <0.05, ** = <0.01, *** = <0.001, **** = <0.0001).

**Fig. 7.**
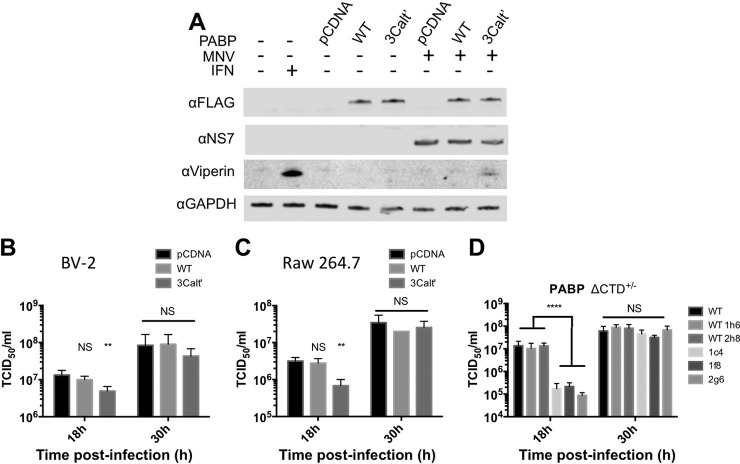
**Modulation of PABP cleavage during MNV infection is inhibitory for viral replication.** (*A*) Western blot analysis of BV-2 cells infected at high MOI (10) with MNV and transfected with wild-type or noncleavable (Q440A) PABP. Viral titers obtained following low MOI (0.01) infection of (*B*) BV-2 or (*C*) RAW 264.7 cells transfected with wild-type or a noncleavable form (Q440A) of PABP. (*D*) Viral titers obtained following low MOI (0.01) infection of cells heterozygous for a truncated form of PABP. Error bars represent standard deviation from three biological replicates. Statistical analysis was performed by one-way ANOVA. (* = <0.05, ** = <0.01, *** = <0.001, **** = <0.0001).

Previous studies with feline calicivirus or recombinant norovirus protease suggested a potential role for the calicivirus 3C-like protease in the cleavage of PABP (29). The biological consequence of the norovirus protease-mediated cleavage was not examined due to the lack of an available cell culture system at the time the study was undertaken. PABP was identified in both whole-cell lysate and m7GTP samples; however, its abundance was largely unmodified. Notably, only a small fraction of PABP cleavage has been shown to be required for effects on host cell translation (44). To determine if the MNV NS6 protease identified in the m7GTP analysis contributed to the loss of eIFs from the eIF4F complex, the ability of NS6 to cleave initiation factors was examined. Expression of a GFP-NS6 fusion protein resulted in the cleavage of PABP, while other initiation factors known to be targets of other 3C or 3C-like proteases remained uncleaved (Fig. 6*B*) (45). Side-by-side comparison of the cleavage products of PABP from 293T cells expressing the MNV receptor (CD300lf, (46, 47)) and either the protease alone or infected with MNV reveals that this cleavage product can be observed in infected cells, though additional cleavage products are also present (Fig. S4*A*). Analysis of time-course samples from infected cells shows the appearance of cleavage products from 9 h post infection, consistent with the timing of the impact on cellular translation (Fig. 6*C*). Not all of the PABP detected by Western blotting was cleaved during infection, in agreement with previous observations for other positive sense RNA viruses where cleavage of a fraction of PABP has an impact on cellular translation (44). The anti-PABP antibody used to examine cleavage during infection detects both PABP1 and PABP3, therefore it was not possible to distinguish if the partial cleavage observed by Western blotting was the result of an isoform specific effect of NS6. The expression of NS6 alone was sufficient to have a small but reproducible and significant impact on cellular translation in the absence of viral replication (Fig. 6*D*), without any obvious impact on cellular viability (Fig. S4*B*). Expression of NS6 alone in mock- or interferon-treated cells confirmed that NS6 expression alone was at least partially responsible for the reduced translation of induced ISGs (Fig. S5*B* and S5*C*). PABP consists of an N-terminal region containing multiple RNA recognition motifs and the eIF4G-binding site, and a C-terminal region containing the PABC-domain (Fig. 6*A*). Many viruses target the flexible linker region connecting these two domains (48). Mutational analysis was used to demonstrate that the NS6 protease cleavage site was Q440, also known as the 3Calt' site (Fig. S5*A*). The biological consequence of PABP cleavage and the impact of cleavage on virus replication was examined by overexpressing either wild-type PABP or a noncleavable (Q440A) form of PABP in MNV permissive cells. The expression of a noncleavable form of PABP resulted in the partial restoration of ISG translation during infection (Fig. 7*A*) as low levels of viperin induction was seen during infection. This partial restoration of ISG induction also resulted in a delayed replication during low multiplicity, multicycle replication typically causing a 1 log_10_ reduction in viral titers at 18 h post infection (Figs. 7*B* and 7*C*).

To investigate whether infection or cleavage of PABP in infected cells impacted on PABP localization, confocal microsocopy was used to examine the localization of PABP in infected cells. Both mock and infected cells possessed diffuse cytoplasmic PABP localization, with no evidence of altered localization in response to MNV-infection apparent (Fig. S6*A*). Attempts were made to use CRISPR-mediated gene editing to generate MNV-permissive cells expressing a noncleavable form of PABPC1 (Fig. S6*B*); however, all clones that were isolated were heterozygous for a PABP C-terminal deletion (all clones possessed a frameshift at P433, resulting in a truncation 20 amino acids later) (Fig. S6*C*). When not infected, cell viability (Fig. S6*D*) and translation (Fig. S6*E*) in these cells remained unaffected. Upon infection, an approximate 100-fold drop in viral titers at 18 h post infection was observed in cells containing the truncated form of PABP (Fig. 7*D*). These data demonstrate a role for NS6 cleavage of PABP in at least partially reducing cellular translation in infected cells.

To examine if NS6-mediated cleavage of PABP also resulted in the observed loss of eIF3 from the eIF4F complex the effect of NS6 cleavage on the recruitment of eIF3 to the eIF4F complex was examined by the enrichment of the eIF4F complex on m7GTP-Sepharose. The recruitment of eIF3D to the eIF4F complex was not affected by PABP cleavage, yet both full length and the N-terminal cleavage products of PABP were recruited to the eIF4F complex (Fig. S5*D*). These data suggested that, while NS6-mediated PABP cleavage plays a partial role in reducing cellular translation in MNV-infected cells, other pathways also contribute. One possible explanation would be the triggering of apoptosis, previously described as being induced by MNV infection (49), which could explain the additional cleavage products of PABP in infected cells (*e.g.* Figs. 6*C* and S4A) as PABP is known to be cleaved during apoptosis (50).

##### Loss of eIF3 Recruitment to Translation Initiation Complexes Is Part of the Cellular Response to Infection

**Fig. 8.**
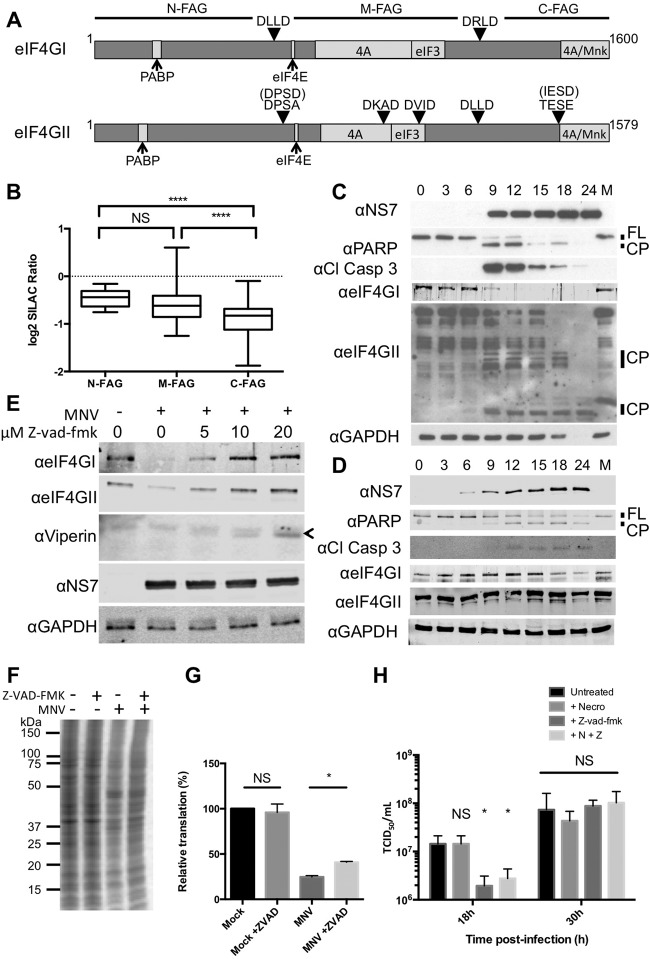
**Induction of apoptosis and caspase cleavage of eIF4F components also contributes to altered translation and alters eIF4F composition.** (*A*) A diagram illustrating the structure of eIF4GI and II as well as their caspase cleavage sites. (*B*) Quantification of peptides mapping to the N-FAG, M-FAG, or C-FAG domains of eIF4GI binding m7GTP-Sepharose beads at 9 h post infection. (*C*) Western blotting against eIF4G and markers of apoptosis for an infection timecourse from BV-2 or (*D*) RAW 264.7 cells. (*E*) Western blotting of eIF4GI or II, and viperin in the presence of varying amounts of the caspase inhibitor z-vad-fmk. The specific viperin band is highlighted with “<” (*F*) ^35^S-Methionine pulse labeling of BV-2 cells mock- or infected with MNV at 9 h post infection in the presence or absence of the z-vad-fmk inhibitor. (*G*). Quantification by phosphorimaging of three biological repeats of experiment shown in (*F*). (*H*). Viral titers obtained following low MOI (0.01) infection of BV-2 cells treated with z-vad-fmk (20 μm), necrostatin-1 (40 μm) singly or in combination. *n* ≥ 3, error bars represent standard deviation. Statistical analysis was performed by one-way ANOVA. (* = <0.05, ** = <0.01, *** = <0.001, **** = <0.0001).

Apoptosis is a key cellular pathway known to inhibit translation initiation (30). MNV infection causes the induction of apoptosis through down-regulation of survivin (51), leading to the activation of a number of caspases as well as cathepsin B (52). The eIF4GI and II proteins are targets for caspase-mediated cleavage at multiple positions as shown in Fig. 8*A* (30). The SILAC quantification presented in Fig. 4 represents an averaged fold-change across all the quantified peptides from an individual protein. In the case of eIF4GI, over 60 peptides from this protein were identified in the m7GTP-Sepharose purified samples across the entire protein, enabling the direct identification of eIF4G fragments that may remain associated with the eIF4F complex if caspase cleavage had occurred. Caspase cleavage of eIF4GI results in the production of three fragments, of which only the middle fragment (M-FAG) would be expected to bind efficiently to m7-GTP Sepharose. Differing amounts of the N-FAG, M-FAG, and C-FAG fragments were found associated with the eIF4F complex at 9 h post infection, consistent with caspase-mediated cleavage resulting in the preferential loss of fragments that do not interact directly with eIF4E (Fig. 8*B*). Retention of N-FAG could be explained as an indirect interaction mediated through PABP and the mRNA. To examine the correlation between induction of apoptosis and the impact of infection on cellular translation, the activation of caspase 3, the cleavage of PARP, eIF4GI, and eIF4GII was examined by Western blotting. In BV-2 cells, loss of full-length eIF4GI and II and the appearance of prominent eIF4GII cleavage products were apparent from 9 h post infection, concomitant with the appearance of cleaved-caspase 3 and PARP (Fig. 8*C*). Loss of the cellular marker GAPDH was also seen at late times, corresponds to a reduction in cell viability (Fig. S7). In contrast, during replication in RAW 264.7 cells the induction of apoptosis was less pronounced and somewhat delayed in agreement with previous observations (11, 49, 51) with only low levels of cleaved caspase being detected and incomplete cleavage of PARP (Fig. 8*D*). The marked cleavage of eIF4GI and II was also not readily observed. Cleaved caspase 3 and PARP cleavage were observed from 9 h post infection; however, unlike in BV-2 cells, levels of cleaved caspase 3 remained low, and PARP cleavage did not reach completion. While the contribution of individual caspases can vary, efficient PARP cleavage is a hallmark of apoptotic cell death. Incomplete PARP cleavage has been previously linked to accelerated cell death through a combination of apoptosis and necroptosis and linked to low intracellular ATP and NAD^+^ levels (53). This suggests that cell death in RAW264.7 cells may not be exclusively apoptotic and offers an explanation as to why the apoptotic phenotype is less pronounced in these cells.

To determine if the cleavage of eIF4GI and II during MNV infection was the result of apoptosis, the effect of the pan-caspase inhibitor z-vad-fmk on eIF4G cleavage was examined. As previous studies have indicated that the inhibition of apoptosis in BV-2 cells results in rapid induction of necroptosis (54), the necroptosis inhibitor necrostatin-1 was included where noted. eIF4GI and II levels were restored by the inhibition of caspases in a dose-dependent manner that also correlated with a partial restoration of translation of the representative ISGs (Fig. 8*E*). The impact of infection on the cellular translation profile of cells was also partially restored when apoptosis was inhibited (Fig. 8*F*), while this effect was slight, quantification of repeat samples by phosphorimaging revealed it was significant (Fig. 8*G*). Furthermore, the inhibition of apoptosis and the resulting increase in the translation of induced ISG mRNAs lead to delayed replication kinetics during a multicycle replication experiment (Fig. 8*H*).

## DISCUSSION

As obligate intracellular pathogens, viruses must replicate within host cells and must therefore balance the use of host resources and the ability to evade/control the cellular response to infection. In this study, we demonstrate that noroviruses alter cellular translation through the modification of translation initiation factors to not only favor viral translation but also impede the translation of genes induced as a result of the innate immune response. This offers a new mechanism by which norovirus is able to regulate the immune response and demonstrates that by modifying translation in infected cells, noroviruses are capable of preventing production of ISGs such as STAT1 and ISG15, known to be important for control of norovirus infection (17, 55).

**Fig. 9.**
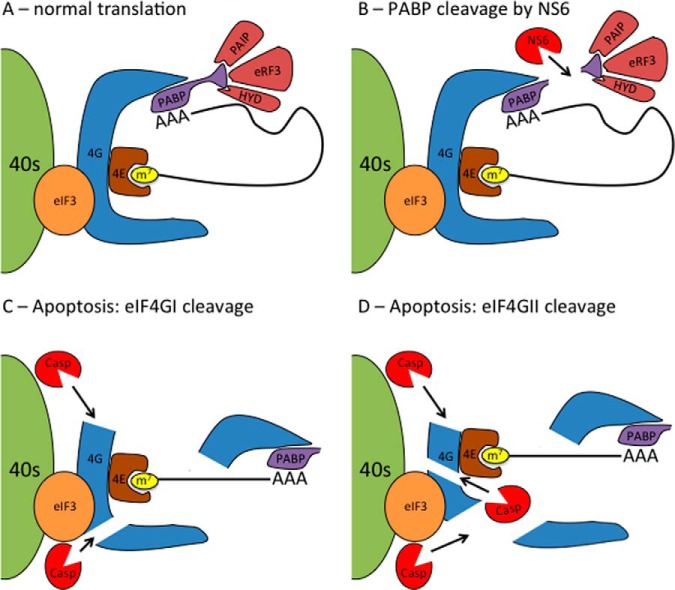
**Model for modifications to eIF4F during norovirus infection.** (*A*) In healthy cells, the 5′ cap of mRNA is bound by eIF4E. This is bound by the scaffolding protein eIF4G that allows binding to other initiation factors (eIF4A, eIF3, PABP) as well as recruitment to the ribosome via the eIF3 complex. The norovirus protease NS6 alone can inhibit translation through cleavage of PABP, though larger-scale alterations to the initiation factor complex are not observed. In infected cells, the induction of apoptosis can result in further modification of the eIF complex with caspase cleavage of (*C*) eIF4GI separating the eIF4E and PABP-binding regions and abolishing the circularization of translating mRNAs. (*D*) Cleavage of eIF4GII by caspases is more extensive and in addition to the effects observed with eIF4GI cleavage, separates the eIF4E and eIF3-binding domains of eIF4GII, preventing recruitment of mRNA to the ribosome.

In the current study, we observed that PABP cleavage by the norovirus protease plays a role in modification of host translation and can impact on the translation of induced ISGs. PABP cleavage has also been observed following infection with a range of viruses, including other caliciviruses (29), picornaviruses, HIV (56, 57), and others (48). The actual mechanism by which PABP cleavage inhibits or modifies cellular translation remains unclear. PABP plays multiple roles within the cell; cytoplasmic PABP is involved in translation enhancement via a bridging interaction with eIF4G leading to a “closed loop” conformation of the RNA that in turns is believed to increase RNA stability and promote ribosome recycling (58). Through additional interactions with the ribosomal release factor eRF3, PABP is also believed to play a role in translation termination, contributing to the control of nonsense-mediated mRNA decay (59, 60). While early research hypothesized that cleavage would prevent mRNA circularization (61), more recent data suggest that the N-terminal region of PABP is necessary and sufficient for both poly(A) and eIF4G-binding (62). The cleavage of PABP by picornaviruses is also incomplete and targets polysome associated PABP (29, 62, 63), indicating the protease targets only a subset of PABP molecules. The C terminus of PABP is required for binding of a number of protein partners, including PAIP1 and 2, and eRF3 (64, 65). It is likely that cleavage inhibits the recruitment of these proteins to actively translating mRNA, leading to reduced ribosome release and recycling of subunits (62). The norovirus NS6-mediated cleavage removes the C-terminal oligomerization domain, which could act in a dominant manner to prevent the extension of PABP oligomers on the poly(A) tail of cellular (or viral) mRNA (66). However, the impact of limited PABP cleavage on the oligomerization of PABP on the poly(A) tail is yet to be fully explored. Of note, our data from heterozygous CRISPR-modified cells show that a mixed population of full-length PABP and the C-terminally truncated PABP fragment are sufficient for growth and normal levels of translation in unstressed/uninfected cells. This suggests that partial recruitment of PABP-binding proteins to polysomes is sufficient for normal protein synthesis; however, more complete loss is inhibitory (Fig. 9*B*).

Caspase-mediated cleavage of initiation factors has multiple effects on cellular translation (67, 68) (69). In the case of eIF4GI, cleavage causes the linearization of the mRNA as the PABP-binding N-FAG fragment of eIF4GI and the middle M-FAG fragment containing the eIF4E and eIF3-binding sites are separated following cleavage (Fig. 9*C*). Apoptotic translation shutoff correlates with cleavage of eIF4GII where additional caspase target sites further degrade eIF4GII and separate the eIF4E and eIF3 binding sites preventing recruitment of the mRNA to 43 s subunits (Fig. 9*D*) (69). Of note, VPg-dependent viral translation appears to continue even after apoptotic cleavage of eIF4G has initiated (Figs. 3 and 8). This is consistent with recent data on VPg-dependent translation where the middle fragment of eIF4G is sufficient for norovirus translation (4, 6). The importance of this late viral translation for virus biology remains to be determined.

The mass spectrometry results successfully identified modification consistent with caspase-cleavage of eIF4G but also yielded further details on alterations to the eIF complex, including diminished eIF4AII, but not eIF4AI levels, and alterations to eIF4B binding. Particularly in the case of eIF4AII, there have been conflicting reports that this specific form of eIF4A is vital for microRNA-mediated translation inhibition (70, 71). Whether the loss of eIF4AII is due to the viral or host response merits further investigation. This work represents the first use of SILAC-based quantitative proteomics to study norovirus infection or virus-mediated translation inhibition. Furthermore, this study makes clear that quantitative proteomics is capable of offering up a new level of detail with regard to identifying the roles and responses of individual eIF components under stress conditions and their contribution to the translation efficiency of individual mRNAs.

Other viruses have been shown to modify eIF components in order to alter cellular translation (16). One example would be African swine fever virus, which utilizes both eIF4E phosphorylation and redistribution of the translational machinery to viral factories in order to control host translation and the immune response (72). However, the best characterized example of eIF4F modification is from the picornaviruses that utilize two viral proteases to create a environment where host translation is completely inhibited (38). The effects observed in norovirus-infected cells in many ways resemble those generated in picornavirus infection, however, there are important distinctions. First, the scale of inhibition seen is clearly different with picornaviruses causing complete host shutoff, while noroviruses merely cause a reduction of host translation (Fig. 3). Secondly, shutoff in picornavirus infection is an early event and allows the exclusive use of host translation apparatus for efficient viral translation (38), whereas in norovirus infection, altered translation is a relatively late event and comes at a time when viral replication appears largely complete (Figs. 1 and 3). While the shutoff observed in picornaviruses would also inhibit production of ISGs in response to infection, in norovirus infection, this would appear to be the primary effect of reduced cellular translation at this time during infection (Figs. 2 and 3). A final distinction is in the mechanism of inhibition, with picornavirus shutoff driven entirely by two viral proteases (2A, 3C) that are necessary and sufficient for the observed phenotype. In contrast, norovirus utilizes just a single viral protease—NS6, a 3C-like protease that, of the initiation factors tested, cleaves only PABP (Fig. 6). However, norovirus infection is capable of inducing further alterations to translation initiation by utilizing the induction of apoptosis and in particular, caspase activation in order to mimic the effects of 2A protease cleavage on eIF4G (Fig. 8) (73). While apoptosis is also induced in poliovirus-infected cells, the contribution of this to eIF4G cleavage is unclear (74). The cells used in this study are macrophage or microglia-like cell lines, and in the infected host, the virus is known to be able to infect dendritic cells and macrophages (75) and, most recently, B cells (76). While the proviral nature of the apoptotic response in norovirus infection has been discussed previously, it is possible that this mechanism plays a role in a subset of infected cells within the host. Of particular note is the recent finding that enteric bacteria may also be able to modify this response, with *Salmonella* coinfection inhibiting MNV-induced apoptosis, diminishing viral replication, and notably also increasing cytokine levels produced in response to infection (77), fitting with our hypothesis that apoptosis is used by noroviruses as a mechanism to suppress the translation of induced ISGs.

In summary this study demonstrates than norovirus infection modifies the ability of host cells to respond to infection by limiting the translation of induced mRNAs. The alterations to host translation observed are induced both directly by the virus, as well as through the induction of apoptosis, and serve to counter the paracrine impact of the innate response.

## DATA AVAILABILITY

The mass spectrometry proteomics data have been deposited to the ProteomeXchange Consortium via the PRIDE (37) partner repository with the dataset identifiers PXD004984 (whole-cell SILAC experiments) and PXD004983 (m7GTP enrichment).

## Supplementary Material

Supplemental Data

## Footnotes

^1^ The abbreviations used are:

eIF: eukaryotic initiation factor
ISG: interferon-stimulated gene
MNV: murine norovirus
MOI: multiplicity of infection
PABP: poly(A) binding protein
RAG: recombination-activating gene
SILAC: stable isotope labelling of amino acids in cell culture
STAT1: signal transducer and activator of transcription 1
PABP: poly(A) binding protein
TCID: Tissue culture infectious dose
ANOVA: analysis of variance
GAPDH: glyceraldehyde 3-phosphate dehydrogenase
PFA: paraformaldehyde
NAT1: Novel APOBEC-1 target 1
CRISPR: clustered regularly interspaced short palindromic repeats
FAG: fragment
PAIP: PABP interacting protein
eRF: eukaryotic release factor.
