# Supplementary material for: Norovirus-Mediated Modification of the Translational Landscape via Virus and Host-Induced Cleavage of Translation Initiation Factors
Source: Mol Cell Proteomics. 2017 Jan 13;16(4 Suppl 1):S215–29. doi: 10.1074/mcp.M116.062448 (PMC5393397; doi:10.1074/mcp.M116.062448)
Supplement: Supplemental Data [file supp_16_4-suppl-1_S215__index.html]

Norovirus-mediated modification of the translational landscape via virus and host-induced cleavage of translation initiation factors — Norovirus-Mediated Modification of the Translational Landscape via Virus and Host-Induced Cleavage of Translation Initiation Factors — Norovirus Control of Host Translation — Supplemental Data 

# Norovirus-Mediated Modification of the Translational Landscape via Virus and Host-Induced Cleavage of Translation Initiation Factors

## Supplemental Data

- Table S1 (.xlsx, 55 KB) - Table S1. STRING analysis of proteins showing a >2-fold change in abundance in the 9h m7G-sepharose pulldown.
- Table S2 (.xlsx, 2.4 MB) - Table S2. Whole cell lysate SILAC data from MNV-infected BV-2 cells.
- Table S3 (.xlsx, 449 KB) - Table S3. m7GTP-sepharose pulldown SILAC data from MNV-infected BV-2 cells.
- Figure S1-S7 (.pdf, 15.1 MB) - Figures S1-S7 and figure legends.
- Table S1-S3 legends (.pdf, 41 KB) - Legends for Tables S1-S3
