# Supplementary material for: Norovirus-Mediated Modification of the Translational Landscape via Virus and Host-Induced Cleavage of Translation Initiation Factors
Source: Mol Cell Proteomics. 2017 Jan 13;16(4 Suppl 1):S215–29. doi: 10.1074/mcp.M116.062448 (PMC5393397; doi:10.1074/mcp.M116.062448)
Supplement: Supplemental Data [file 10.1074_M116.062448_mcp.M116.062448-4.pdf]

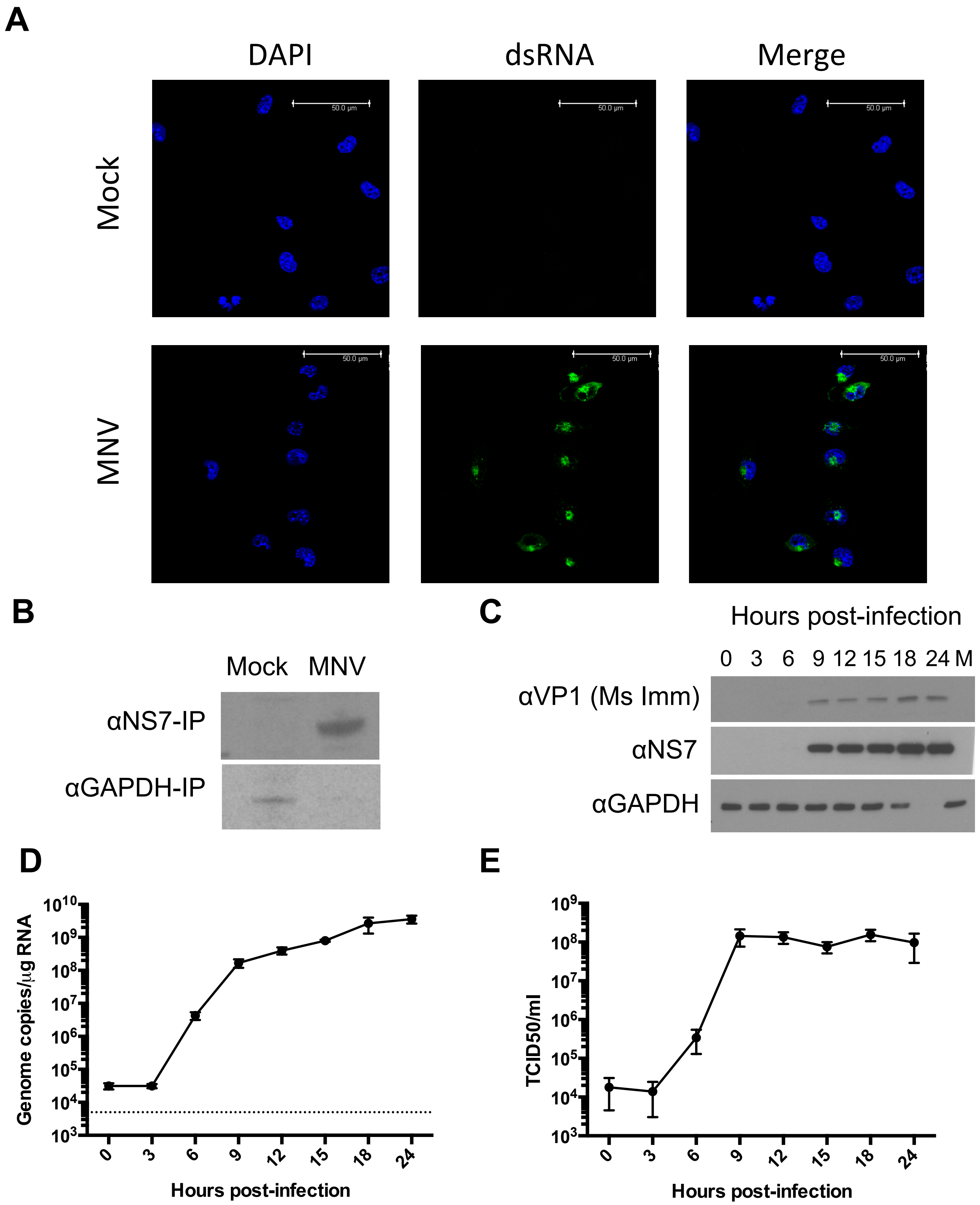
A
DAPI
dsRNA
Merge
Mock
MNV
50.0 µm
B
Mock MNV
αNS7-IP
αGAPDH-IP
C
Hours post-infection
0 3 6 9 12 15 18 24 M
αVP1 (Ms Imm)
αNS7
αGAPDH
D
Genome copies/µg RNA
10^{10}
10^9
10^8
10^7
10^6
10^5
10^4
10^3
0 3 6 9 12 15 18 24
Hours post-infection
E
TCID50/ml
10^9
10^8
10^7
10^6
10^5
10^4
10^3
0 3 6 9 12 15 18 24
Hours post-infection

Figure S1. **Translation at late times post-infection is dominated by viral proteins** A) Immunofluorescence using anti-dsRNA confirms the infection conditions used (MOI 10) result in infection of a majority of BV-2 cells. B) Immunoprecipitation of viral (NS7) and cellular (GAPDH) proteins from S35-labelled infected BV-2 cells at 12hpi reveals translation of viral, but not cellular proteins. Analysis of a high multiplicity of infection timecourse in BV-2 cells shows viral C) protein, D) genome copies and E) titres have largely peaked by 9 hpi.

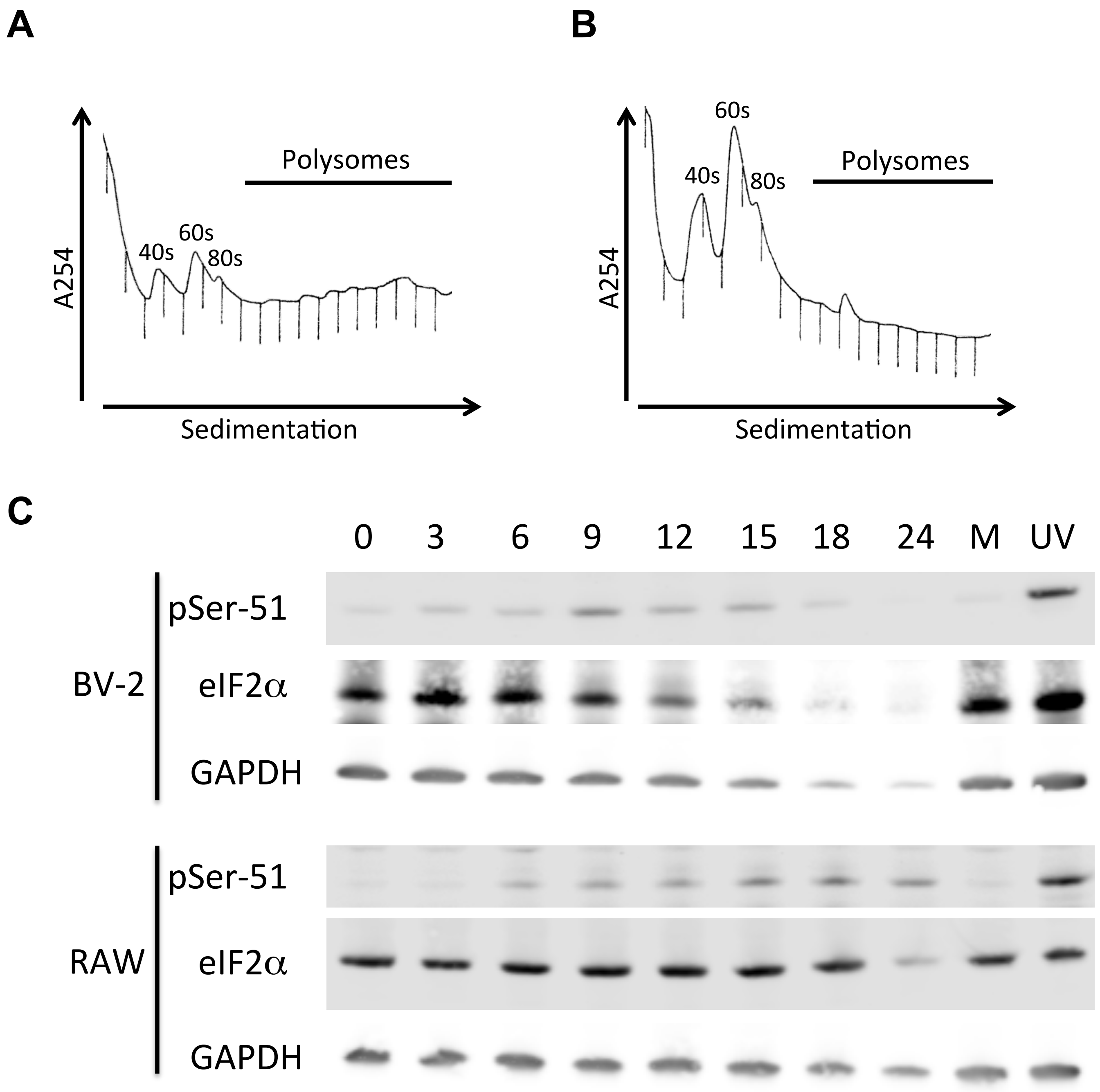
A
B
A254
40s
60s
80s
Polysomes
Sedimentation
C
0
3
6
9
12
15
18
24
M
UV
BV-2
pSer-51
eIF2α
GAPDH
RAW
pSer-51
eIF2α
GAPDH

Figure S2. **A defect in translation inhibiton occurs without high eIF2α phosphorylation** Polysome profiling of A) Mock or B) MNV-infected BV-2 cells performed under high salt conditions (400mM KCl) to separate 80s monosomes into 40s and 60s subunits. C) Western blot analysis of eIF2α phosphorylation in MNV-infected RAW 264.7 or BV-2 cells.

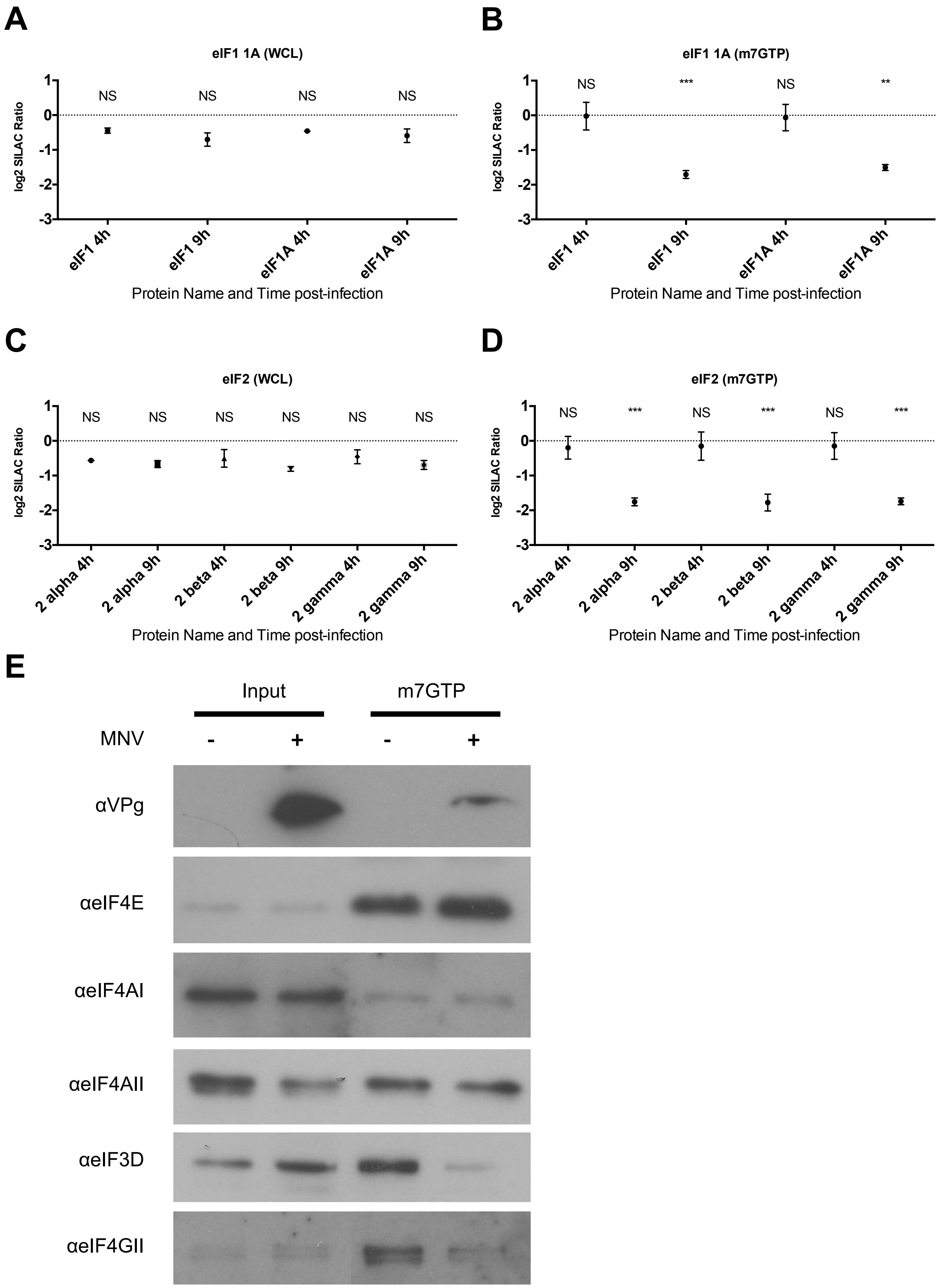
A
eIF1 1A (WCL)
B
eIF1 1A (m7GTP)
C
eIF2 (WCL)
D
eIF2 (m7GTP)
log2 SILAC Ratio
NS
***
**
eIF1 4h
eIF1 9h
eIF1A 4h
eIF1A 9h
2 alpha 4h
2 alpha 9h
2 beta 4h
2 beta 9h
2 gamma 4h
2 gamma 9h
Protein Name and Time post-infection
E
Input
m7GTP
MNV
αVPg
αeIF4E
αeIF4AI
αeIF4AII
αeIF3D
αeIF4GII

Figure S3. eIF changes upon MNV-infection and validation by western blotting. Downstream of eIF3, additional initiation factor components also show diminished binding to m7GTP-sepharose at late times following MNV-infection, including A-B) eIF1, and C-D) eIF2. E) Western blotting analysis of selected initiation factor binding to m7GTP-sepharose identified in mass spectrometry analysis. Significance was tested by 1-way ANOVA comparing changes to a control protein with unaltered abundance (eIF4E). (*=<0.05, **=<0.01, ***=<0.001, ****=<0.0001). Where a protein was identified in only a single mass spectrometry replicate, precluding statistical analysis, this is indicated with 'N.D.', otherwise proteins were identified in at least 2/3 replicates..

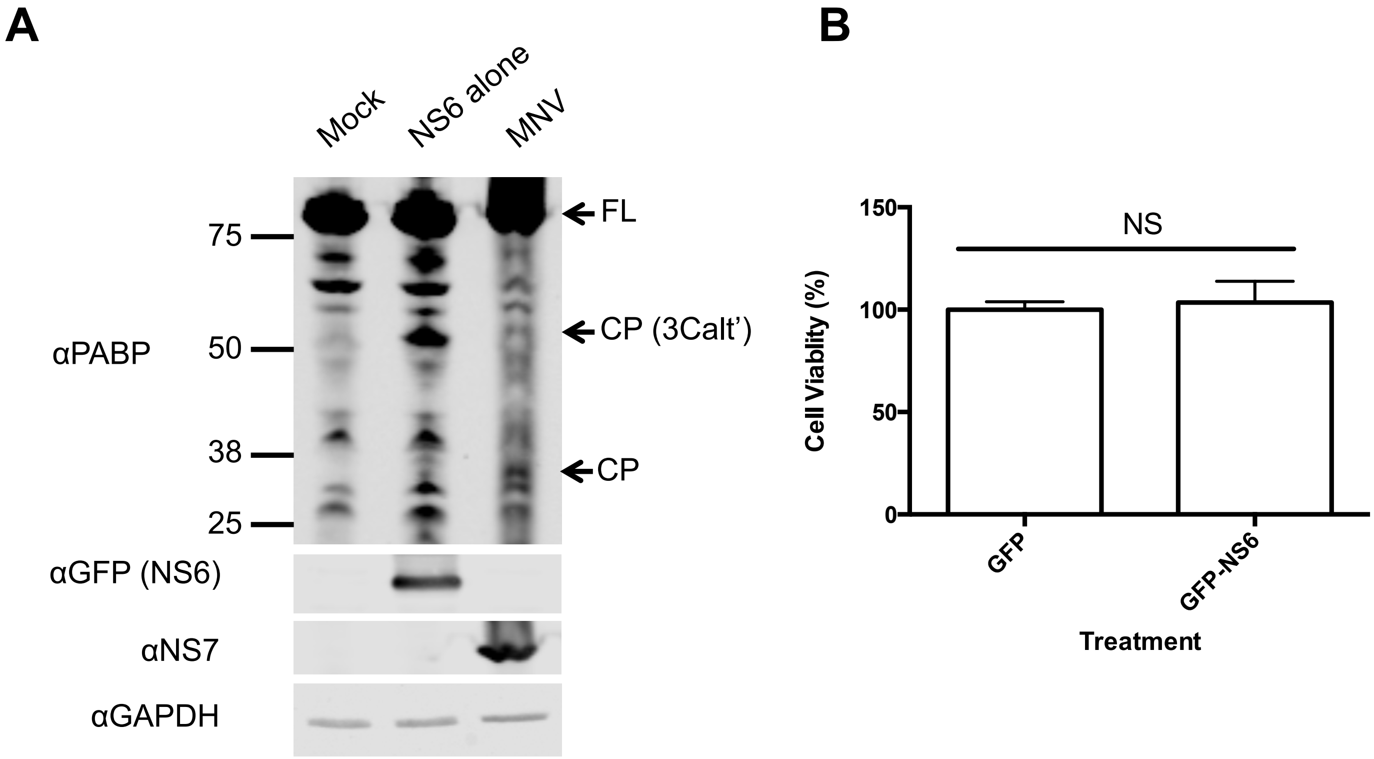
A
Mock
NS6 alone
MNV
FL
75
CP (3Calt')
αPABP
50
38
CP
25
αGFP (NS6)
αNS7
αGAPDH
B
150
NS
100
Cell Viability (%)
50
0
GFP
GFP-NS6
Treatment

Figure S4. Comparison of PABP cleavage by NS6 and during MNV infection. A) Western blot analysis of 293T cells stably expressing the MNV receptor and either mock transfected, transfected with GFP-NS6, or infected at MOI 10 with MNV. Full length or cleaved PABP are arrowed and indicated by 'FL' or 'CP' respectively. B) Cell viability assay using Cell Titre Blue in cells transfected with pEGFP-C1 NS6 confirms NS6 expression does not impact cell viability during the first 24h post-transfection.

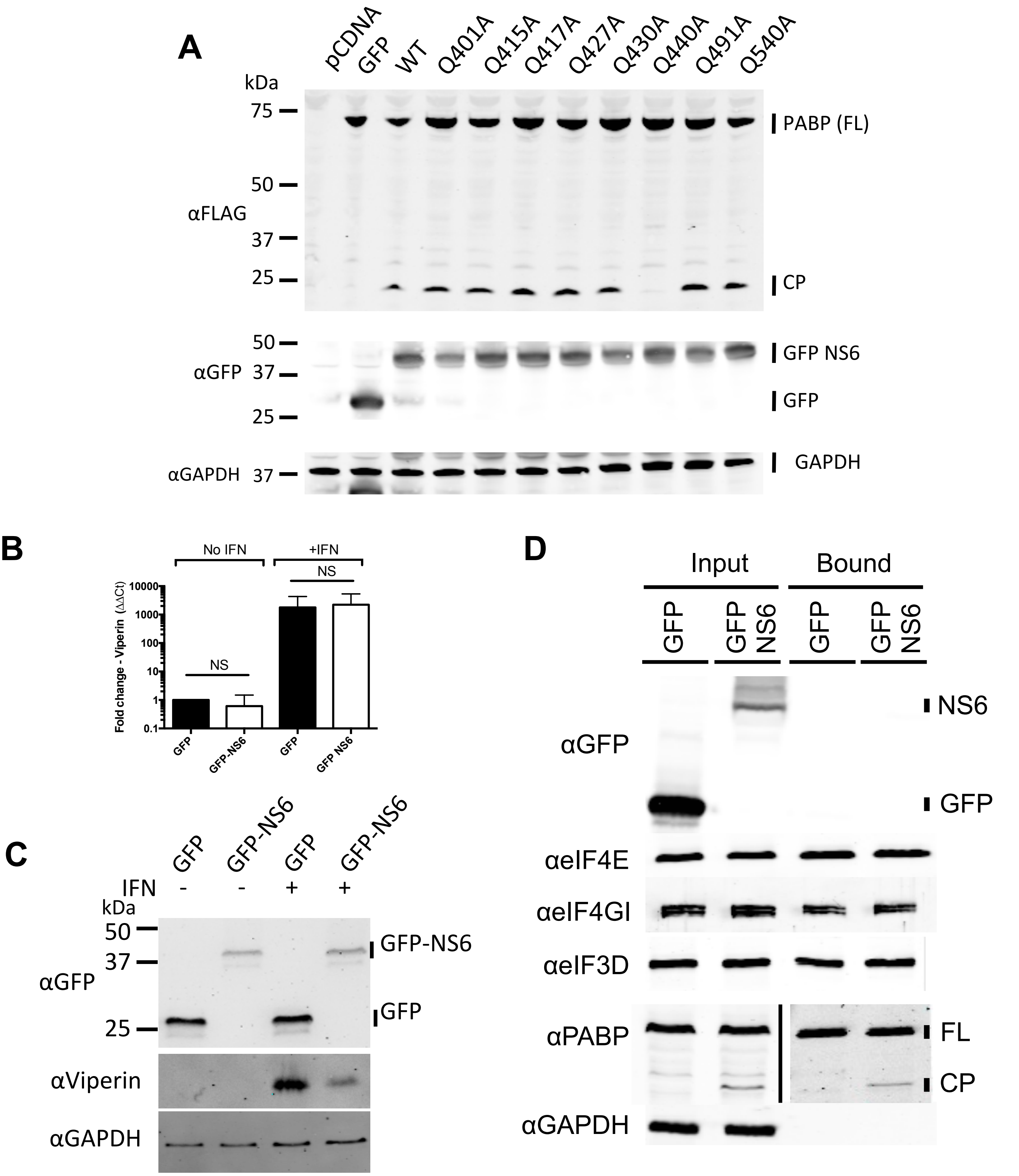

A
pCDNA
GFP
WT
Q401A
Q415A
Q417A
Q427A
Q430A
Q440A
Q491A
Q540A
kDa
75
50
37
25
αFLAG
PABP (FL)
CP
αGFP
GFP NS6
GFP
αGAPDH
GAPDH
B
No IFN
+IFN
NS
Fold change - Viperin (ΔΔCt)
10000
1000
100
10
1
0.1
GFP
GFP-NS6
GFP NS6
C
GFP
GFP-NS6
IFN
-
+
kDa
50
37
25
αGFP
GFP-NS6
GFP
αViperin
αGAPDH
D
Input
Bound
GFP
GFP NS6
αGFP
NS6
GFP
αeIF4E
αeIF4GI
αeIF3D
αPABP
FL
CP
αGAPDH

Figure S5. Identification of the site of PABP cleavage by NS6 and contribution to eIF complex modification and phenotype. A) Western blot analysis of FLAG-tagged WT or mutant PABP identifies Q440 as the NS6 cleavage site. B) qRT-PCR or C) western blot analysis of the levels of the ISG Viperin in pEGFP-C1 NS6 transfected 293T cells D) Western blot analysis of m7GTP-sepharose pulldowns from 293T cells transfected with pEGFP-C1 NS6. Error bars represent standard deviation. Differences in mRNA levels were not significant by one-way ANOVA.

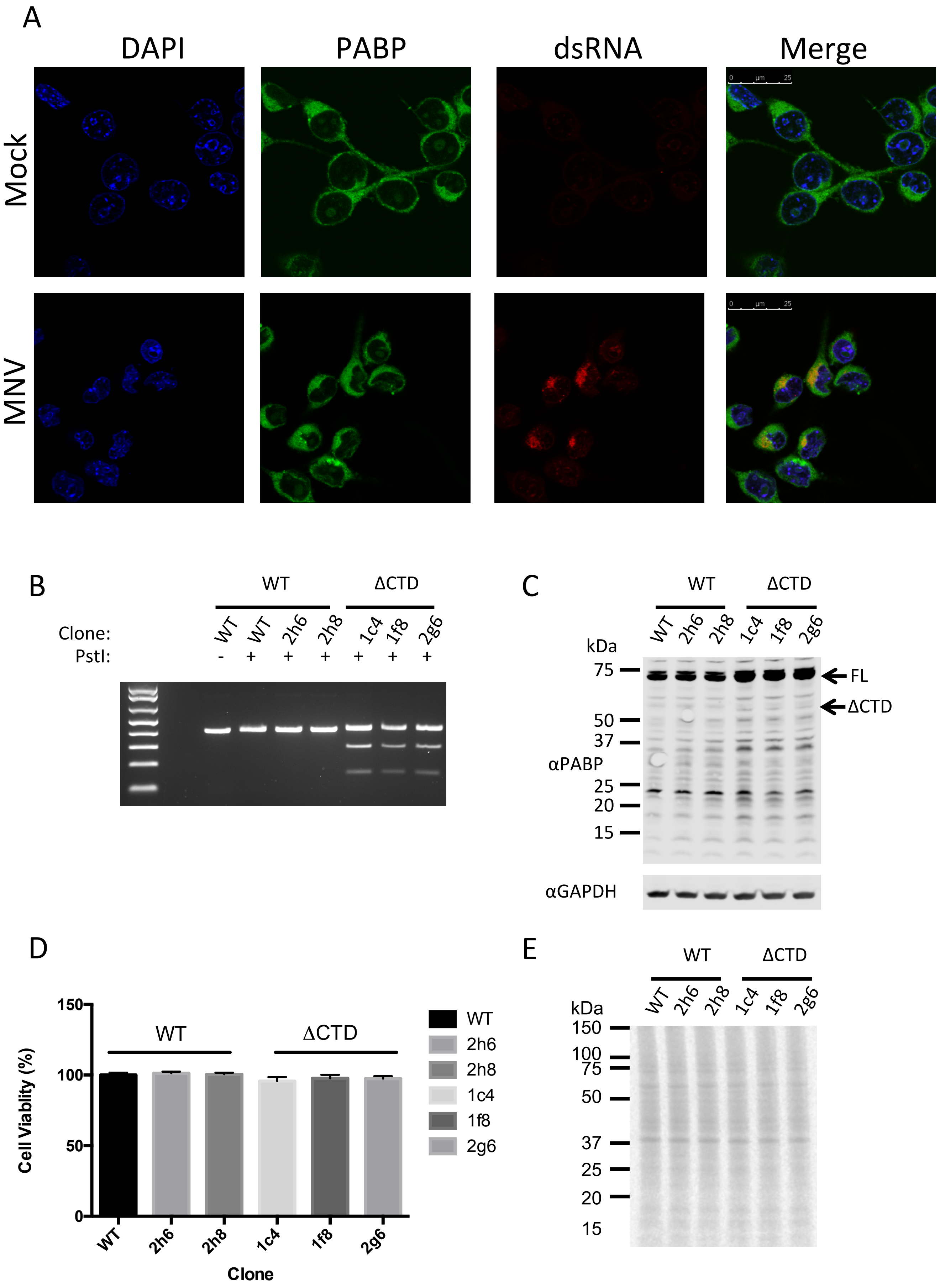
A
DAPI
PABP
dsRNA
Merge
Mock
MNV
B
WT
ΔCTD
Clone:
PstI:
WT
WT
2h6
2h8
1c4
1f8
2g6
C
kDa
75
50
37
25
20
15
αPABP
FL
ΔCTD
αGAPDH
D
Cell Viability (%)
150
100
50
0
Clone
E
kDa
150
100
75
50
37
25
20
15

Figure S6. Confocal microscopy of PABP localization & generation of PABP ΔCTD (+/-) BV-2 A) Confocal microscopy of BV-2 cells at 12h post-infection with MNV showing no alteration in PABP localization. B) Amplicons from cells heterozygous for a PABP ΔCTD mutation contained a *Pst*I restriction site introduced by homologous recombination. C) Western blot analysis of wild-type or PABP ΔCTD. D) Cell titre blue cell viability assay and E) translation from uninfected wild-type or PABP ΔCTD heterozygous BV-2 cells, as determined by autoradiography of 35S-methionine pulsed cells was comparable.

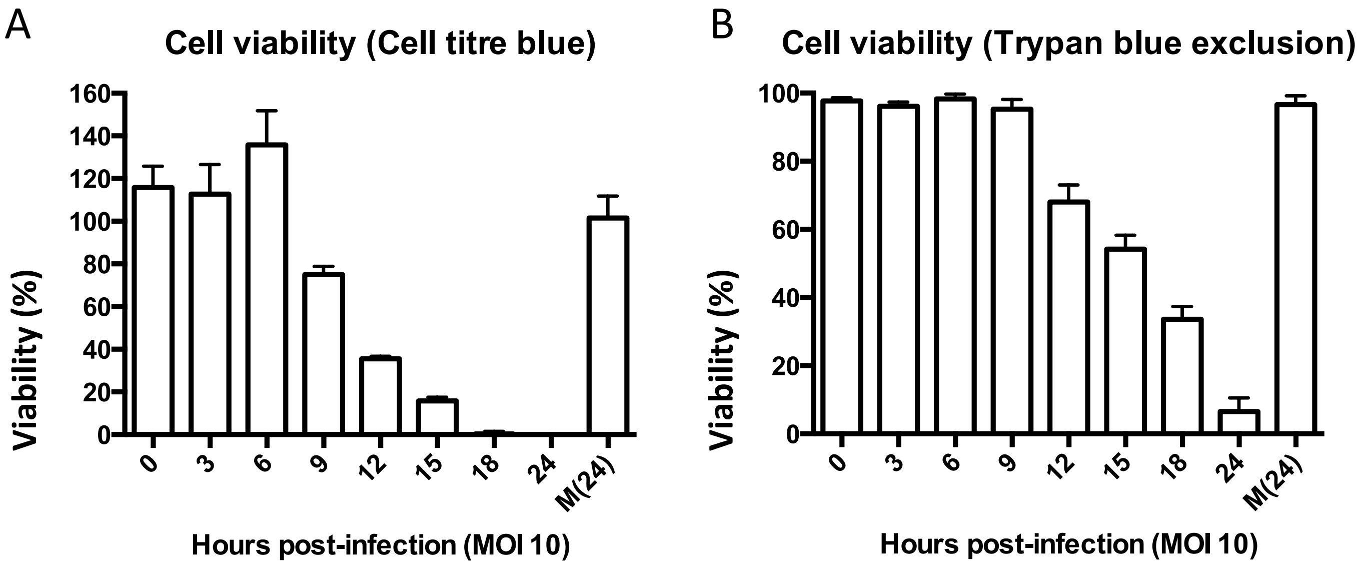
A
Cell viability (Cell titre blue)
Viability (%)
0
20
40
60
80
100
120
140
160
0
3
6
9
12
15
18
24
M(24)
Hours post-infection (MOI 10)
B
Cell viability (Trypan blue exclusion)
Viability (%)
0
20
40
60
80
100
0
3
6
9
12
15
18
24
M(24)
Hours post-infection (MOI 10)

Figure S7. MNV infection reduces cell viability at late times post-infection. Cell viability was monitored by A) Cell titre blue or B) trypan blue staining. Trypan blue staining can be accomplished immediately at the time point indicated, cell titre blue staining requires several hours to develop which could explain discrepancies between the two methods. Error bars indicate standard deviation.
