# Supplementary material for: Norovirus-Mediated Modification of the Translational Landscape via Virus and Host-Induced Cleavage of Translation Initiation Factors
Source: Mol Cell Proteomics. 2017 Jan 13;16(4 Suppl 1):S215–29. doi: 10.1074/mcp.M116.062448 (PMC5393397; doi:10.1074/mcp.M116.062448)
Supplement: Supplemental Data [file 10.1074_M116.062448_mcp.M116.062448-5.pdf]

Table S1. STRING analysis of proteins showing a ≥2-fold change in abundance in the 9h m7G-sepharose pulldown. Proteins showing a arbitrary >2-fold alteration in m7GTP-sepharose binding were inputted into the STRINGv10 database (42). Enriched GO Processes, Molecular Functions, and Cellular compartments are shown on different tabs.

Table S2. Whole cell lysate SILAC data from MNV-infected BV-2 cells. Protein and peptide identifications and quantification from analysis of whole cell lysates from BV-2 cells infected with MNV at 0h, 4h and 9h post-infection are shown. SILAC data from the three experimental repeats are shown. For experiments 1 and 3: L represents Mock, M represents 4h and H represents 9h post-infection. In Experiment 2 the M and H samples are switched. Columns list the uniprot accession numbers for each protein group, the number of peptides and unique peptides identified for each protein group, sequence coverage (%), Maxquant score, protein ratios and gene ontology annotations. Additional columns detailing the number of peptides identified in each experiment, the number used for quantification, and the variability in quantification are included as hidden columns for brevity, but can be unhidden if required. Proteins of interest shown are highlighted in green. Full unmodified data including the ability to view individual spectra are available as part of PRIDE submission PXD004984.

Table S3. m7GTP-sepharose pulldown SILAC data from MNV-infected BV-2 cells. Protein and peptide identifications and quantification from analysis of m7GTP-enriched samples prepared from BV-2 cells infected with MNV at 0h, 4h and 9h post-infection are shown. For experiments 1 and 3: L represents Mock, M represents 4h and H represents 9h post-infection. In Experiment 2 the M and H samples are switched. SILAC data from the three experimental repeats are shown. Columns list the uniprot accession numbers for each protein group, the number of peptides and unique peptides identified for each protein group, sequence coverage (%), Maxquant score, protein ratios and gene ontology annotations. Additional columns detailing the number of peptides identified in each experiment, the number used for quantification, and the variability in quantification are included as hidden columns for brevity, but can be unhidden if required. Proteins of interest are highlighted in green. Full unmodified data including the ability to view individual spectra are available as part of PRIDE submission PXD004983.
